# A Systematic Review to Compare Electrical, Magnetic, and Optogenetic Stimulation for Peripheral Nerve Repair

**DOI:** 10.1016/j.jhsg.2024.03.005

**Published:** 2024-06-29

**Authors:** Priya Kaluskar, Dhruv Bharadwaj, K. Swaminathan Iyer, Christopher Dy, Minghao Zheng, David M. Brogan

**Affiliations:** ∗ Centre for Orthopaedic Research, Medical School, The University of Western Australia, Nedlands, WA, Australia; † Perron Institute for Neurological and Translational Science, Perth, Australia; ‡ ARC Training Centre for Personalised Therapeutics Technologies, Department of Biochemistry and Pharmacology, School of Biomedical Sciences, University of Melbourne, Melbourne, Australia; § Medical School, The University of Western Australia, Nedlands, WA, Australia; ‖ School of Molecular Sciences, the University of Western Australia, Perth, Australia; ¶ ARC Training Centre for Next-Gen Technologies in Biomedical Analysis, School of Molecular Sciences, the University of Western Australia, Perth, Australia; ∗∗ Orthopaedic Surgery Division of Hand and Microsurgery, Department of Orthopaedic Surgery, Washington University School of Medicine, St. Louis, MO; †† Department of Orthopaedic Surgery, Washington University School of Medicine, St. Louis, MO

**Keywords:** Electrical stimulation, Magnetic stimulation, Optogenetic stimulation, Peripheral nerve repair

## Abstract

The purpose of this systematic review was to assess the currently available evidence for the use of external stimulation to modulate neural activity and promote peripheral nerve regeneration. The most common external stimulations are electrical stimulation (ES), optogenetic stimulation (OS), and magnetic stimulation (MS). Understanding the comparative effectiveness of these stimulation methods is pivotal in advancing therapeutic interventions for peripheral nerve injuries. This systematic review focused on these three external stimulation modalities as potential strategies to enhance peripheral nerve repair (PNR). We used the Preferred Reporting Items for Systematic Reviews and Meta-Analyses framework to systematically evaluate and compare the efficiency of ES, OS, and MS in PNR. The review included studies published between 2018 and 2023 using ES, OS, or MS for PNR focused on enhancing recovery of peripheral nerve injuries in rodent models identified through PubMed and Google Scholar. The search strategies and inclusion criteria identified 19 studies (13 ES, 4 OS, and 2 MS) for detailed analysis, focusing on critical parameters such as functional recovery, histological outcomes, and electrophysiological data. Although ES demonstrated a consistent improvement in all the analyses, high-frequency repetitive MS (HFr-MS) emerged as a promising modality. HFr-MS demonstrated accelerated PNR, as histological and electrophysiological evidence indicated. In contrast, OS exhibited superior functional recovery outcomes. Notable limitations include constrained MS and OS data sets and the challenge of comparing relative improvements because of methodological diversity in evaluation techniques. Our findings underscore the potential of HFr-MS and OS in PNR while emphasizing the critical need for standardized testing protocols to facilitate meaningful cross-study comparisons. External stimulations have the potential to improve functional recovery in patients with nerve injury.

The prevalence of peripheral nerve injuries (PNIs) is around 2.2% in patients with trauma, increasing to 5% when brachial plexus injuries and root avulsions are considered.^1^, ^2^, ^3^ Peripheral nerve injuries can result in chronic pain, numbness, muscle weakness, and diminished range of motion, often leading to considerable and sometimes permanent disability, whereas the inherent limitations of the natural regeneration process contribute to suboptimal functional recovery.^4^ Rehabilitation through nonpharmacological and noninvasive stimulation therapy has been explored to improve functional recovery in patients with PNIs. The most commonly used stimulation therapy method is electrical stimulation (ES), but other stimulation strategies, such as magnetic stimulation (MS) and optogenetic stimulation (OS), have recently been investigated.^5^, ^6^, ^7^

Electrical stimulation can be administered through implantable bioelectric devices, wearable and portable stimulation devices, closed-loop systems, nanotechnology, or nanoelectrodes.^8^^,^^9^ A combination of external stimulation and nerve reconstruction surgery can accelerate muscle reinnervation and enhance neuronal survival and axonal sprouting when applied directly following crush, transection, and long-distance injuries.^10^, ^11^, ^12^, ^13^, ^14^, ^15^, ^16^ The effects of ES have been known for centuries.^17^ Hoffman^18^ postulated that ES leads to the bombardment of antidromic signals to the neuron’s cell body, accelerating axoplasm synthesis. The intracellular pressure increases because of the increase in axoplasm, leading to forward extrusion and axonal sprouting.^18^ Electrical stimulation has shown promise in accelerating PNR by enhancing neuronal survival, axonal sprouting, and increasing levels of cyclic adenosine monophosphate and nerve growth factors in dorsal root ganglions and Schwann cells, respectively.^19^ Gradual release of cyclic adenosine monophosphate enhances regenerative capacity in peripheral nerves.^20^, ^21^, ^22^, ^23^

Magnetic stimulation is a noninvasive method to stimulate neuronal growth.^24^ Several in vitro studies on neuronal cells show that MS can potentially influence the outgrowth of neurites and neuronal interaction. In cases of neuropathic pain, repetitive transcranial magnetic stimulation (TMS) has been shown to have an analgesic effect on the patients effectively. Magnetic stimulation has been shown to increase the expression of anti-inflammatory and reduce proinflammatory cytokines, highlighting its crucial role in neuroinflammation.^25^, ^26^, ^27^ Often, the factors that determine the efficiency of MSs are treatment size, frequency of the stimulations, and interaction between the frequency and lesion of the stimulation site.^28^ In clinical scenarios, repetitive TMS is widely used in 5–10 Hz in the primary motor cortex area at 80% to 90% resting motor threshold and for 5–10 treatment sessions to produce analgesic effects. Magnetic stimulation shows a potential for decreasing proinflammatory cytokines and increasing the expression of anti-inflammatory cytokines. It has also been shown to regulate neuronal tissue inflammation and protective abilities against apoptosis.^29^

Optogenetic stimulation is a newer mode of stimulation compared with MS and ES. Optogenetic stimulation uses G-coupled membrane proteins called opsins (first discovered as halo rhodopsin by Oesterhelt and Stoeckenius [1971]) to regulate cellular activities. Opsins are of two types—microbial opsins (including type I rhodopsin and ion transport proteins) and animal opsins (type II rhodopsin and G-protein-coupled receptors and melanopsin).^7^^,^^30^, ^31^, ^32^, ^33^ Among these, microbial opsins are mainly used to regulate cellular activities, and animal opsins are used as optogenetic tools for neuronal excitability.^34^^,^^35^ However, OS is not limited to photoexcitation or photoinitiation; instead, it can lead to gain or loss of function. However, its relatively slow temporal precision, multicomponent character, and need for high-intensity ultraviolet light represent significant limitations to its widespread use.^36^, ^37^, ^38^, ^39^ Moreover, achieving sufficient expression and regulation of the opsin proteins without compromising specificity is challenging.^40^

ES, OS and MS can be compared over a wide range of aspects to distinguish and highlight their application and potential of impact on PNR (Table 1). This systematic review explored these three external stimulation modalities—ES, OS, and MS as strategies to enhance PNR. By analyzing in vivo advancements of these external stimulations, this review seeked to identify, introduce, and compare them to gain insights into their effectiveness and potential clinical application in PNR.

**Table 1:** Detailed Comparison of Optical, Magnetic and Electrical Stimulation for Peripheral Nerve Repair Strategies

Aspect	Optogenetics	Magnetic Stimulation	Electrical Stimulation
Mechanism of Action	Uses light-sensitive proteins (opsins)^41^	Uses strong magnetic fields with/without coils depending on transcranial/transcutaneous use^42^, ^43^, ^44^	Applies electrical currents through the use of micro (electrodes)^45^
Targeted Stimulation	Precise, cell-specific^41^^,^^46^	Moderate selectivity (uncertain to some extent), however dependent on methods applied with TMS navigation and/or reduction in coregistration errors, limited knowledge about TNS^47^, ^48^, ^49^	Broad, affecting nearby tissues due to neo-currents^45^^,^^50^
Depth of Penetration	Limited to the surface – though it can be increased with spectrally shifted variants^51^	Can penetrate deeper depending on coil design^43^^,^^44^^,^^52^	Can penetrate deep into tissues^45^^,^^50^^,^^53^^,^^54^
Invasiveness	Generally minimally invasive^55^	Non-invasive^47^^,^^52^^,^^56^	Invasive (electrodes required)^45^^,^^53^^,^^54^
Temporal Precision	Highly precise milliseconds^41^^,^^55^^,^^57^	Precise, but slower response^52^^,^^58^	Milliseconds to seconds^59^^,^^60^
Tissue Heating	Minimal heat generation^61^	It can produce heat in the brain through the use of magnetic power and impedance^52^	Can generate heat locally^45^^,^^59^
Safety Concerns	There is a low risk of tissue damage; however, devices to deliver light, like implanted optical fibres aimed at deep targets, can displace or damage local tissues^62^	Generally safe (for transcranial magnetic resonance); however, safety and efficacy are not well understood with coils due to insufficient data^52^^,^^63^^,^^64^	Risk of tissue damage and burns^45^^,^^59^
Applications	Neuroscience, cell research, cell – mechanobiology^55^^,^^65^	Brain and muscle stimulation^48^^,^^52^^,^^64^	Neuromuscular stimulation, pain, neuromodulation and neuro synapticity^45^^,^^54^^,^^59^
Limitations	Limited to genetically modified cells and specific wavelength of light^61^	Depth limitations, however, coil diameter and magnetization force controllable factors in influencing depth^48^^,^^52^^,^^64^	Potential tissue damage^45^^,^^59^
Precision of Activation	Precise spatial and temporal control^41^^,^^57^^,^^61^	Spatially precise, less temporal^47^^,^^48^^,^^52^^,^^58^	Spatially and temporally less precise^59^^,^^60^^,^^66^
Side Effects	Minimal side effects in target cells; however, variable cell response if other cells are stimulated and thus can be difficult to control^67^	Few side effects, some discomfort depending on intensity^47^^,^^48^^,^^52^^,^^56^^,^^58^	Potential for side effects like pain^45^^,^^60^
Genetic Modification	Requires genetic modifications^65^	No genetic modifications needed^42^^,^^52^^,^^56^	No genetic modifications needed^45^^,^^54^^,^^60^
Scalability	It can be challenging to scale up due to the ethical concerns around modifying gene expression and influencing localized cell response. Cost is also a relevant factor.^68^^,^^69^	Scalable to larger areas (however issues with cost and insufficient data currently available)^42^^,^^48^^,^^52^^,^^56^	Scalable to different intensities and pulse frequencies depending on the required focus with the required peripheral nerve interface^45^^,^^50^^,^^54^^,^^60^
Regulatory Approval	Potential challenges due to genetic manipulation^68^^,^^69^	Approved for various medical uses^52^	Approved for various medical uses^45^^,^^50^^,^^54^^,^^60^
Reversibility	Reversible with light on/off or changing intensity/wavelength of the stimulation light rays can dictate the influence of ligand-based proteins^61^^,^^62^^,^^65^^,^^68^	Reversible with stimulation on/off^43^^,^^52^^,^^63^	Reversible with current on/off^45^^,^^70^
Control of Excitation	Excites neurons with light, but control/focus reduces as intensity falls exponentially^41^^,^^51^^,^^61^^,^^69^	Induces action potentials proprioceptive afferents when applied to muscles via two pathways. The first pathway is the direct activation of sensorimotor nerve fibres. The other is the indirect activation of mechanoreceptors in the muscle fibre and some effects due to increased blood inflow^52^^,^^71^	Induces action potentials^45^^,^^60^^,^^72^
Compatibility	Compatible with specific cell types such as cardiovascular and neurite cells/axons where there can be control of the ligand-based membrane channel proteins^41^^,^^51^^,^^61^^,^^69^	Compatible with neural tissue^52^^,^^71^	Compatible with various tissues, specifically nerve and skeletal muscle tissue; however, increasing usage in retinal research^45^^,^^59^^,^^60^^,^^72^
Research vs. Therapy	Primarily used in research but can also be used in cardiovascular research/PNI^51^	Used in both research and therapy^52^^,^^71^	Used in both research and therapy^45^^,^^59^^,^^60^^,^^72^
Targeted Diseases	Used to study neural circuits^55^^,^^73^^,^^74^	Various neuropsychiatric disorders^48^^,^^52^^,^^58^^,^^71^	Neuromuscular diseases, pain^45^^,^^59^^,^^60^^,^^72^
Imaging Compatibility	Compatible with imaging techniques^65^	May interfere with imaging due to the superposition and interference effects of neighbouring magnetic fields due to induced currents^48^^,^^52^^,^^58^^,^^71^	Compatible with imaging techniques^45^^,^^50^^,^^59^^,^^60^^,^^72^

## Materials and Methods

This systematic review followed the Preferred Reporting Items for Systematic Reviews and Meta-Analyses guidelines.^75^

### Search strategy

A comprehensive literature search was conducted using PubMed and Google Scholar from September 27, 2023 to October 4, 2023. The inclusion criteria comprised research published between 2018 and 2023, written in English, and involving studies on rats or mice models. The filters used were “other animals” in species.

### Inclusion criteria

1. Only original full-text studies were considered.
2. Studies exclusively involving rats or mice were included.
3. Only English publications were included.
4. Studies specifically included sciatic nerve injuries that assessed electrical, magnetic, or optogenetic stimulations.
5. Only studies with appropriate controls were included.
6. Studies published between 2018 and 2023 were included in the review.

### Exclusion criteria

1. Review articles and case studies were excluded from the study.
2. Studies with no controls were eliminated.
3. Studies on nerves other than sciatic nerves were excluded.
4. Studies that did not use stimulation (ES, MS, or OS) for regeneration were excluded.
5. Studies that did not include in vivo analysis of outcomes in rat or mouse models were excluded from the review.
6. Studies unavailable in the English language were excluded.
7. The analysis did not include studies focusing on using stimulation for pain management.

### Study key words and identification

For ES, Google Scholar was systematically searched using the following key words: electrical stimulation peripheral nerve regeneration axonal repair “rat” OR “mice,” “electrical stimulation” -central -magnetic -optogenetic -dog, -rabbit. A PubMed search included the terms “electrical stimulations AND peripheral nerve repair” with filters “last five years,” “other animals,” and “English.”

Similarly, for OS we conducted Google Scholar and PubMed searches using the following key words: optogenetics in PNIs or axon regeneration -magnetic -electroceutical -drosophila -systematic -zebrafish; “optogenetics stimulation, rats, peripheral nerves,” with filters “English,” “other animals,” and “5 years.”

Google search for MS included the following key words: Nerve Injury AND magnetic stimulation AND control AND in vivo AND Peripheral AND rats repair OR regenerate -Review -book -nanotechnology -electric -humans -mice -patients -Optogenetics, with filters 2018–2023. A PubMed search used the terms (((magnetic stimulation) AND (peripheral)) AND (nerve injury)) AND (rats) with a filter for the last 5 years.

### Selection of articles

The selection of articles involved three stages—title-based selection, abstract-based selection focusing on *in vivo* studies, and a third stage considering nerve injury type, intervention, sample size, and monitoring techniques for regeneration.

### Comparison of simulations

To facilitate a meaningful comparison, outcomes from the studies were systematically analyzed and categorized into quantitative histology, functional analysis, and electrophysiological studies. For each comparison, only studies with each specific test were included. These have been summarized into tables for reference.

Quantitative histology includes aspects of analysis such as the number of neuronal cells in a specific area, the diameter of cells or structures, the number and density of fibers, myelin thickness, and g-ratio. Our review compared axonal counts, myelinated fiber counts, myelin thickness, and axonal diameter. Electrophysiological data included tests such as nerve conduction velocity (NCV) and compound muscle action potential (CMAP). For functional analysis, the most commonly used outcome test. Sciatic function index (SFI) was assessed. SFI analyzes aspects such as toe spread, distance between the footsteps, print length, and distance between the toes.

For statistical comparisons, the fold changes were calculated for each study by comparing the experimental and control groups. Because of a lack of consistency in time points, various time points were compared simultaneously. The differences between the time points and the type of injuries studied in each case have been acknowledged, considered, and discussed in the results.

### Addressing biases and assessing data quality

P.K. and D.B. independently analyzed each set of data for inclusion or exclusion. Discrepancies were resolved by consulting a third author and corresponding author M.Z. and D.B. or through detailed discussion to reach a consensus.

## Results

### Study characteristics

**Figure 1:**
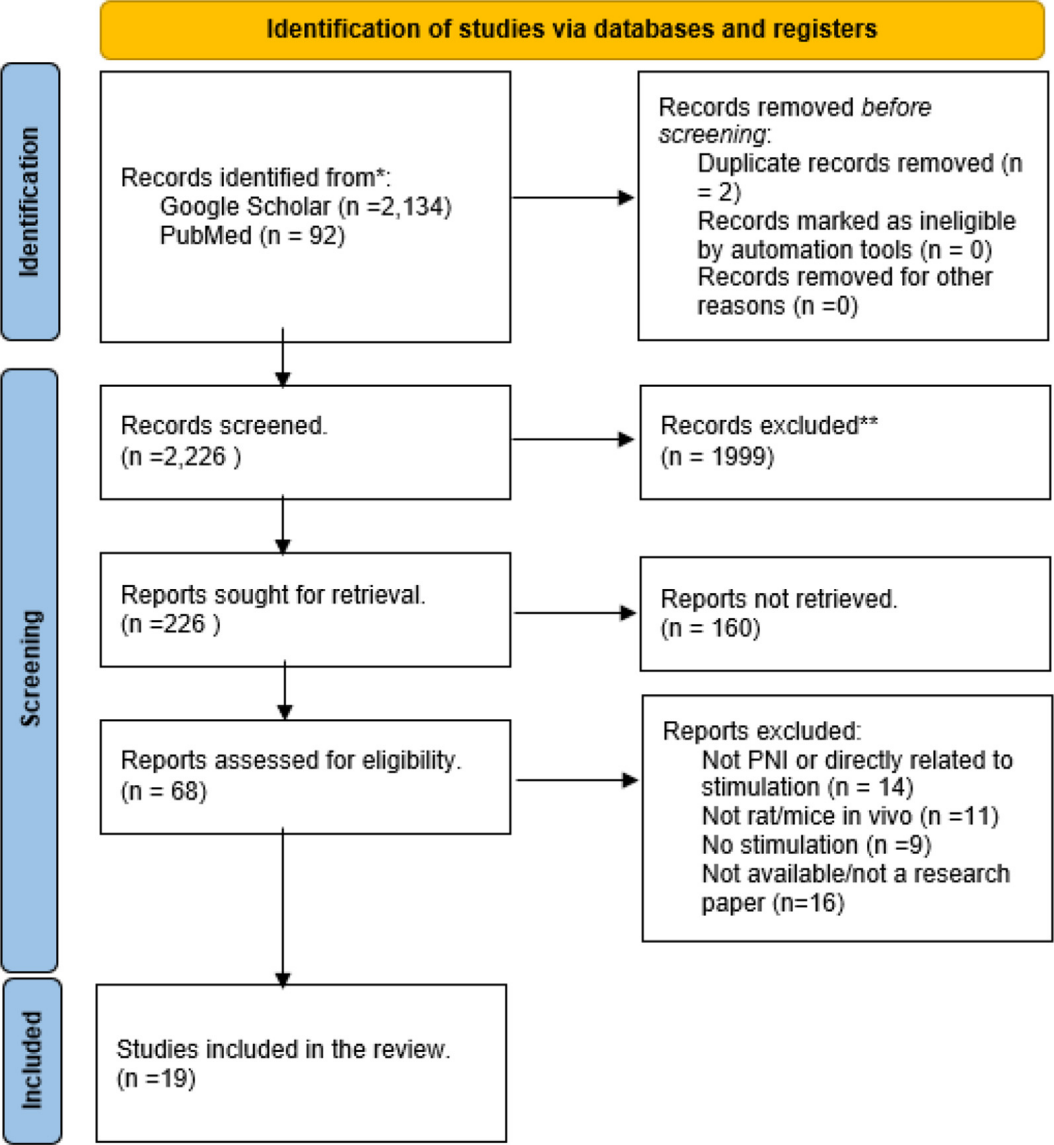
Flow diagram of the process of data selection for the systemic review according to the Prisma framework.^41^

More than 2,000 studies on electrical, magnetic, and optogenetic stimulations were sourced from Google Scholar and PubMed databases and were narrowed to 266 studies based on the inclusion and exclusion criteria. Further scrutiny narrowed the initial selection to 19 studies that met predefined criteria (Fig. 1). This subset comprised 13 studies centred around ES, 4 on OS, and 2 on MS for PNIs in small animals (Table 2). Notably, all these studies were published within the last 5 years (2018–2023).

**Table 2:** The Studies Included as the Final Dataset for This Systematic Review to Compare ES, MS and OS. The Studies Published Between 2018-2023 That Performed *In Vivo* Assessment in Rat or Mouse Model Were Included in the Review

Sr. No.	Title	Aim	Mode of Stimulation	Parameters of Stimulation	Injury	Follow-Up	Sample Size	Control	Monitoring Tests	Results
1	Investigating the effects of brief electrical stimulation duration on sciatic nerve regeneration and functional recovery in a rat transection model^92^	Brief ESs with different durations were compared.	Delivered through two Ag-coated Cu positive electrodes on the proximal part and negative on the distal parts.	Voltage-controlled square pulses characterized by 100 μs width, 20 Hz frequency, and 3V amplitude.	6 mm sciatic nerve gap repaired by a silicon tube	4, 6, 8, 10, 12 weeks	6 rats per group of 10 min, 30 min, and 60 mins stimulation.	No ES	Hot plate test, histomorphometry evaluation	Increase in regeneration speed due to ES. The 60-minute group had better outcomes in histomorphometry assessments.
2	Effect of Intraoperative Electrical Stimulation on Recovery after Rat Sciatic Nerve Isograft Repair^76^	Combined isograft repair in rat sciatic nerve injury to evaluate the effects of intraoperative ES.	Platinum electrodes at proximal and distal ends of the coaptation sites	24 V/m-DC ES for 10 mins	Sciatic gap injury and isograft repair followed by 10 ES before wound closure.	2, 4, 6, 8, 10, 12 weeks	19 for control, 20 for ES	No ES, Sham surgery on the contralateral leg	12 rats were euthanized for histomorphometry analysis at 6 weeks. Extensor postural thrust test, walking track analysis, thermal sensory testing of 8 remaining rats for 12 weeks.	ES showed improvement in function until 12 weeks, with a higher push-off response—no improvement in sensory testing due to ES.
3	Multiple sessions of therapeutic electrical stimulation using implantable thin-film wireless nerve stimulators improve functional recovery after sciatic nerve isograft repair^99^	To determine the optimal time for stimulation to maximize functional recovery of rat sciatic nerve in an isograft repair model.	Thin-film wireless nerve stimulator made of a receiver coil and silicon cuff joined by platinum/iridium wires	ES by transmitter coil output of 200 μs, 50 ms sinusoidal wave, 5 MHz. Inductive coupling induced ES characterized by 3V monopolar square pulses of 200 μs at 20 Hz	Sciatic gap injury and isograft repair	2–18 weeks biweekly	8 rats per group	No ES	CMAP, evoked muscle force, wet muscle mass, axon counting	6 daily sessions of ES were found to be most effective for augmenting functional recovery compared to other time courses of stimulation.
4	Conductive Composite Fibers with Optimized Alignment Guides Neural Regeneration under Electrical Stimulation^93^	The synergistic influence of aligned topography and electrical stimulation on neural regeneration is demonstrated using conductive composite fibres.	Platinum wire electrodes were placed in the proximal and distal ends.	Pulsed current of 20 Hz, 50% duty cycle, 100 mV for 2 hours, 5 times everyday	Sciatic nerve defect of 1 cm, electrospun fibres rolled into NGCs in the experiment group.	12 weeks	5 groups of 9 rats	Autograft suturing distal end with proximal end. and vice versa	Walking track analysis, electrophysiological assessment, immunofluorescence staining, histopathological assessment, muscle weight	Optimized conductive NGCs might serve as good candidates to promote regeneration.
5	Bridging peripheral nerves using a diacetyl chitin conduit combined with short-term electrical stimulation.^95^	To investigate the combination of diacetyl chitin conduit and electrical stimulation for peripheral nerve repair	deacetyl chitin conduit bridging combined with electrical stimulation through trimix Linus apparatus. Two copper wires coiled electrodes secured at the proximal end of the injury.	0.1 ms, 3 V, 20 Hz, for 1 hour	Sciatic nerve transection of 1 cm, 2 mm gap left	6 and 12 weeks	8 rats per group	No ES	Histomorphometry and electrophysiological study	According to histology and electrophysiology data, regeneration at 6 and 12 weeks is better than control.
6	Brief Electrical Stimulation Accelerates Axon Regeneration and Promotes Recovery Following Nerve Transection and Repair in Mice^77^	To determine if a 10-minute ES could improve peripheral nerve repair	A stainless-steel electrode was hooked around the sciatic nerve 2 mm proximal to the repair site. Return electrode in subcutaneous tissue.	16 Hz, 0.5 mA 10 mins or 60 mins	Sciatic nerve transection 2 mm proximal to trifurcation and suture repaired	24 hours, 7 days, 14 days, 56 days	3 groups. 20 each for endpoints and 5 for gene analysis	No ES	Gene expression, histological studies, functional analysis, and relative muscle weight	ES showed increased regeneration-associated genes and improved behavioural recovery—no difference between 10-minute and 60-minute stimulation groups.
7	Transcutaneous and Direct Electrical Stimulation of Mouse Sciatic Nerve Accelerates Functional Recovery After Nerve Transection and Immediate Repair^97^	To investigate and compare transcutaneous and direct electrical stimulation (TCES and DES) to accelerate nerve recovery	A bipolar hook electrode was installed proximal to the injury	1-hour stimulation at 20 Hz with 0.1 millisecond pulse duration at 5V for DES. For TCES, the cutaneous electrodes were placed on the skin at the proximal region. A grass stimulator was used with 90% of the motor threshold for 1 hour.	Sciatic nerve axotomy	Weekly 1–12 weeks	Mice: 4 groups, 4 shams, 8 for axotomy, DES and TCES	Sham and axotomy followed by neurorrhaphy	Sciatic nerve functional recovery and electrophysiology	Functional recovery was better from 8 weeks of DES and TCES. TCES is minimally invasive.
8	Effects of Repeated 20-Hz Electrical Stimulation on Functional Recovery Following Peripheral Nerve Injury^78^	To investigate if repeated Application of ES would be more effective in promoting peripheral nerve regeneration than a single application.	A cuff electrode was placed on the sciatic nerve, and stainless-steel wires were implanted in muscles for EMG. All wires from implants led subcutaneously to the connector plug mounted on the animal's head	20 Hz, 1 hour, 0.3 ms duration pulse for one group and every 3^rd^ day for 2 weeks for another group	Sciatic nerve transection and repair by end-to-end anastomosis using fibrin glue.	0–12 weeks, every 2 weeks	4 mice per group	Same injury but untreated with ES and intact mice	Electrophysiology and histomorphometry	Direct muscle responses increased in ES. Repeated ES did not enhance the rate of restoration of the peripheral nerve.
9	Application of conductive Ppy/SF composite scaffold and electrical stimulation for neural tissue engineering^96^	Composite conductive scaffold fabricated by 3D bioprinting and electrospinning combined with electrical stimulation assessed for PNR.	Two insulated copper wires bared of 3–5 mm insulation were used as electrodes. One tip was looped to secure the proximal and distal ends of the scaffold before implantation. Other wires are connected to the ES device.	Week square 0.1 ms anodic pulses of 3V, 20 Hz applied through skin electrode for 1 hour, every two days seven times.	A scaffold in experiment groups repaired a 10 mm long defect in the sciatic nerve.	2,12,24 weeks	6 rats per group	Autograft and no ES	In vivo studies included sciatic function index (SFI), Real-Time Quantitative Reverse Transcription PCR (qRT-PCR), immunofluorescence staining of tissue and western blot for protein analysis.	Conductive scaffolds with ES promote nerve regeneration and functional recovery.
10	Electrical stimulation accelerates Wallerian degeneration and promotes nerve regeneration after sciatic nerve injury^79^	To explore the effects of brief low-frequency ES on Wallerian degeneration and the mechanisms promoting early nerve regeneration after ES.	The distal nerve end was treated with ES for one hour using a biphasic current pulse, with two electrodes 5 mm apart and the positive electrodes closer to the distal nerve stump, followed by subsequent closure of incisions using 4–0 sutures. In the repair model, ES was performed 5 mm distal to the silicone tube using a biphasic current pulse—a transaction model in which no wire electrodes or ES treatment served as the control group.	A Biphasic current pulse (20 Hz, 100 μs) for one h. The voltage value for each rat was adjusted to just above that required to induce a visible gastrocnemius twitch (mostly a pulsed voltage of 0.2–0.3 V) after the transection of the sciatic nerve.	The sciatic nerve was cut in the middle, transecting approximately 2 mm of tissue, or a gap of 6 mm was created	1, 4, 7, 14 and 21 days post-injury	88 rats, two groups, five-time points. *N* = 4 per group, silicone tube segments at 14 days n = 4 per group and 21-day *N* = 5 per group	No ES	qRT-PCR, histomorphology analysis	Evaluation of nerves bridged using silicone tubing after transection showed that ES promotes nerve regeneration by accelerating wallerian degeneration and upregulating the expression of neurotrophic factors.
11	CNT/Sericin Conductive Nerve Guidance Conduit Promotes Functional Recovery of Transected Peripheral Nerve Injury in a Rat Model^98^	Developing and applying NGC with electrical conductivity and reinforced mechanical properties to repair a 10 mm transected sciatic nerve defect with electrical stimulation.	For the NGC groups, the proximal and distal nerve stumps were secured up to 0.5 mm into the conduits with surgical sutures; then, the rats underwent electrode placement with immediate ES for one hour (No ES for shams).	20 Hz frequency of electrical pulses of 0.1 ms duration at the supramaximal voltage (3 V) for one h.	10 mm sciatic nerve defect	8 and 12 weeks for histological analysis	N = 4 rats per group at 8 weeks and N = 6 rats per group at 12 weeks	Sham, Autologous nerve graft	Histology analysis, SFI, Thermal withdrawal latency (TWL) test, Electrophysiological analysis, and Target muscle analysis.	The conduit and ES promoted structural and function recovery of the sciatic nerve after a 10 mm injury. The outcomes were found to be comparable to autografts.
12	Comparative effects of implanted electrodes with differing contact patterns on PNR and functional recovery^80^	This study evaluated the regenerative effects of implanted electrodes with different contacts in the resected sciatic nerve.	Point contact, 1/4 circle contact; whole-circle contact; no electrodes as control.	Frequency, 20 Hz; Pulse width, 100 μs; Voltage, 9 V; Waveform, square form. ES was conducted 30 min per day for 20 days.	Sciatic nerve resection	4, 8, 12 weeks	48 rats in four groups (n = 12 per group) by electrode contact pattern (n = 6/group per time point).	Control had no electrode.	ES was performed, and electrophysiological, morphological, and histological exams (of the sciatic nerve and muscle).	Electrodes with point and 1/4 circle contacts represented an effective method of electrical stimulation to facilitate injured sciatic nerve regeneration. Whole circle performance was good.
13	Preparation of carboxylic graphene oxide-composite polypyrrole conduits and their effect on sciatic nerve repair under electrical stimulation^94^	The study developed a composite conduit with stable conductivity to evaluate the efficiency of ES in repairing peripheral nerve injuries.	Composite conductive conduit connected with Au threads from the back of the spine	Continuous 20 Hz with a supramaximal pulse (1V, 0.1 ms) for 1 hr/day for a week. The electric field's direction was parallel to the conduit's central axis.	10 mm nerve gap	4, 8, 12 weeks	27 rats, 3 groups, N = 3	Autograft and no ES groups	Histological evaluation and morphological analysis, electrophysiology,	The recovery of nerve s in the ES groups was better than in the no ES groups.
Optogenetics										
1	Bioluminescent Optogenetics: A Novel Experimental Therapy to Promote Axon Regeneration after Peripheral Nerve Injury^88^	To analyse the outcomes of using bioluminescent optogenetics for peripheral nerve regeneration	Excitatory luminopsins were expressed in the motoneurons of transgenic mice. Intraperitoneal administration of coelenterazine (CTZ) to generate bioluminescence in peripheral axons. This bioluminescence increased motoneuron excitability.	After wound closure immediately either CTZ treatment or similar volume of a CTZ solvent (Fuel) control was administered	Sciatic nerve transection and repair by fibrin glue.	4 weeks	60 rats, for in vivo bioluminescence. The sciatic nerve was cut and surgically repaired in 80 mice by fibrin glue mixture.	eLMO3 Mutant R115A used as negative control in contralateral sciatic nerves	M response recovery, retrograde labelling of motoneurons to analyse regeneration after 4 weeks	More motoneurons had successfully reinnervated in targeted muscle 4 weeks after nerve injury in BL-OG-treated mice compared to the controls.
2	Enhancing Motor and Sensory Axon Regeneration after Peripheral Nerve Injury Using Bioluminescent Optogenetics^87^	To study the effectiveness of BL-OG for axonal regeneration following PNI. After retrograding viral transport and neuronal transduction, injected sciatic nerves were cut mid-thigh and repaired by superficial end-to-end anastomosis.	Nerves injected with 1–2 μL of an adeno-associated (AAV2/9) viral vector encoding either an excitatory luminopsin (eLMO3) (1.2 × 10^14^ vg/mL) (4 mice) or a luminopsin with a mutated opsin component (R115A) (3.5 × 10^14^ vg/mL)	Immediately after the repair surgery, the mouse was administered a single dose of CTZ (10 mg/Kg, i.p.)	Sciatic nerve transection and repair	4 weeks	8 mice per group	The contralateral sciatic nerve in each mouse was not injected or injured and served as an intact control	Immunohistochemistry of tissue, electrophysiology	Compound muscle action potentials (M waves) recorded in response to sciatic nerve stimulation were more than fourfold larger in mice expressing the excitatory luminopsin than in controls expressing the mutant luminopsin
3	Optical Stimulation and Electrophysiological Analysis of Regenerating Peripheral Axons^81^	To evaluate activation to promote regeneration by targeted opsin expression to different neuron types Using fibre optics	Position the fibre optic cable so the tip gently contacts the sciatic nerve.	Blue light (473 nM wavelength) to activate channel rhodopsin 2.	Transaction of sciatic nerve and repair by fibrin glue	4 weeks	-	Intact mice	Electrophysiology, latency, EMG, ENG, response amplitude, M responses.	Improved regeneration was noticed in optic fibre stimulation compared to those without stimulation.
4	Optogenetically enhanced axon regeneration: motor versus sensory neuron-specific stimulation^82^	To evaluate the precision and selectivity of system-specific neuronal activation to enhance axon regeneration in a mixed nerve	The fibre optic cable was positioned with a tabletop clamp and maintained in gentle contact with the sciatic nerve immediately proximal to the branching of the three terminal nerves (joint peroneal, tibial, and sural). The optical fibre was attached to a custom-built light source consisting of a laser LED and a collimator that delivered light pulses at 473 nm wavelength under computer control of intensity and duration.	For one hour, brief pulses of blue light (473 nm) were shown directly onto the nerve via a fibre optic cable (200 μm core diameter)	Sciatic nerve transection and repair by fibrin glue	4 weeks	7 intact mice for control and n = 3 or 7 for other study groups	Untreated injury repaired with fibrin glue	Electrophysiology, retrograde labelling, neuromuscular junction analysis	The number of ChR2-positive neurons whose axons had regenerated successfully was more significant following system-specific optical treatment
Magnetic stimulation										
1	Effect of the combination of high-frequency repetitive magnetic stimulation (HFr-MS) and neurotrophin on injured sciatic nerve regeneration in rats^83^	This study sought to observe the effects of HFr-MS neurotrophin and its combined use in treating peripheral nerve injury.	HF-rMS was delivered using a 70 mm coil connected to a magnetic stimulator. The stimulation site was at the intersection of the notochord and proximal end of the reconstructed sciatic nerve. The rat was placed in a customized cloth bag when receiving stimulation.	In the HFr-MS group, peripheral high-frequency repetitive magnetic stimulation treatment (20 Hz, 20 min/d) was delivered ten consecutive days after autografting.	Sciatic nerve Transaction	17 days	32 total, n = 8 for histology	The control group received only a reversed autograft in the left sciatic nerve without treatment.	Basso-Beattie-Bresnahan (BBB) locomotor rating scale, The sciatic functional index (SFI), histology and Immunohistochemistry staining	The number of myelinated fibres on the distal end was 155 in MS, while 100 in control.
2	Stimulation and Repair of Peripheral Nerves Using Bioadhesive Graft-Antenna^90^	The study aimed to use an original wireless stimulator for peripheral nerves based on a metal loop (diameter ≈1 mm) that is powered TMS stimulator	TMS induced by magnetic coil, loop antennae and chitosan conduit	TMS delivered pulsed stimuli at a field magnitude of ≈0.72 T	Sciatic nerve defect of 10 mm	8 weeks	Total 30 rats N = 5 per group in CMAP and 6 in histomorphology	Unoperated contralateral nerves	Histology and electrophysiology analysis	The graft antenna is stable over the nerve and does not shift position or diminish its stimulation capacity over three months.

The 13 ES studies explored diverse parameters, including stimulation duration, mode, and nerve guidance conduits (NGCs). Nerve guidance conduits are hollow cylindrical devices that are wrapped around the nerve injury to help reconnect the injured nerve over a short distance (<30 mm).^84^ Four OS studies ventured into bioluminescence optogenetics and axonal regeneration facilitated by fiber optics. The two MS studies, TMS and repetitive high-frequency MS (HFr-MS), used distinct approaches. These studies were compared based on histological, electrophysiological, and functional recovery outcomes.

### Quantitative histology

Quantitative histology compared axonal counts, myelinated fiber counts, myelin thickness, and axonal diameter.

**Figure 2:**
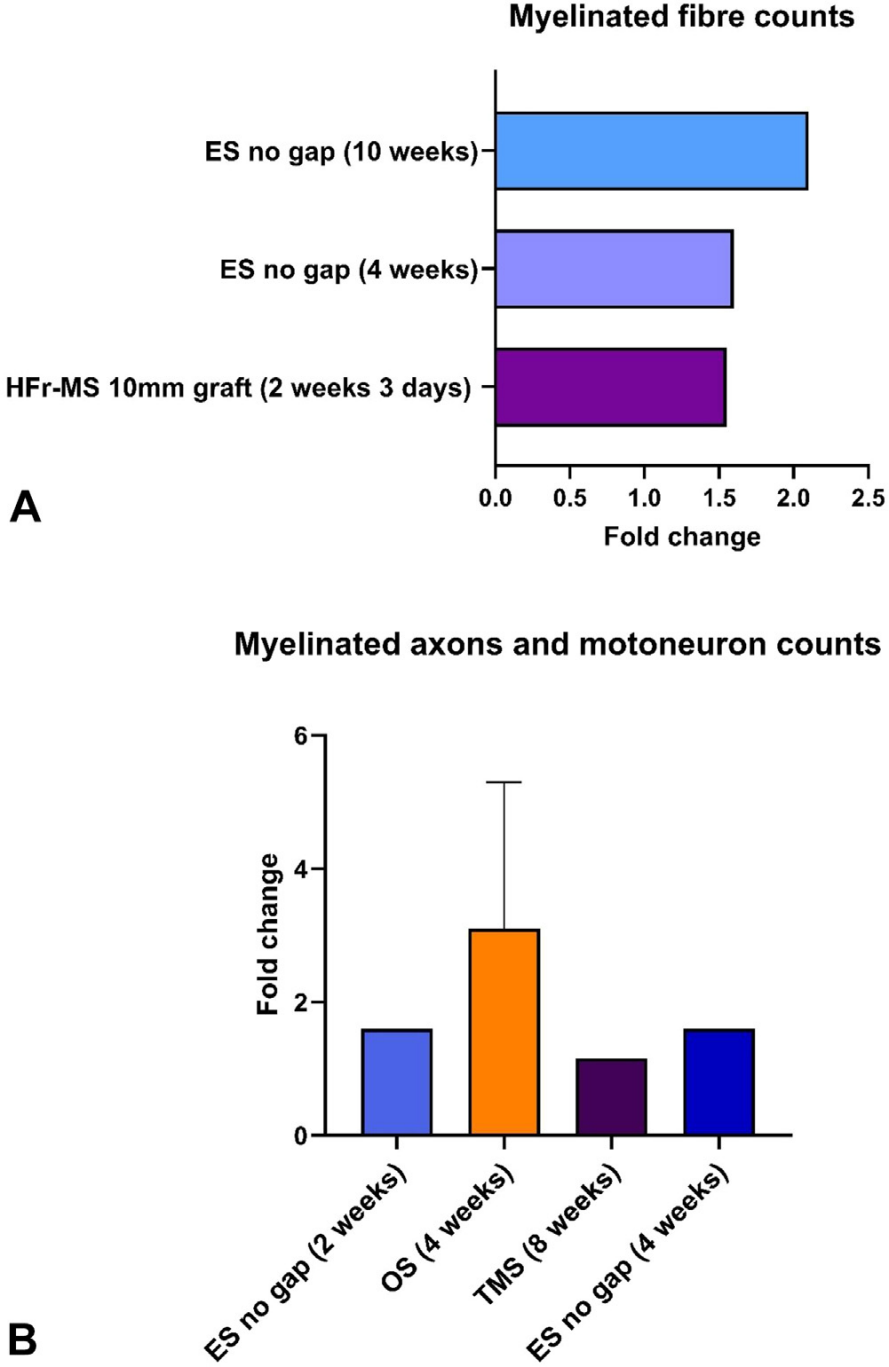
Histomorphometry improvements in the myelinated features such as fibre counts, and axon counts. **A** The fold change in the number of myelinated fibres observed on the distal end of the regenerated nerves in rat/mouse models after various stimulations, indicating the improvement at the tissue level.^85^^,^^86^**B** The fold change in the number of myelinated axons and motoneurons in art/mouse models following various stimulations, indicating improvement at the cellular level.^86^, ^87^, ^88^, ^89^, ^90^

Myelinated fiber counts were analyzed to compare the effect of stimulations (Fig. 2A). The studies included in this analysis are summarized in Table 3. Electrical stimulation observed a 1.6-fold increase in fiber counts at 4 weeks, whereas HFr-MS observed the same increase at around 2 weeks. At a later time point of 10 weeks, ES outperformed HFr-MS by a 2.1-fold increase in fiber count. It is important to note that although outcomes of MS were at par with ES, the follow-up for HFr-MS was earlier and indicates a faster increase in the number of distal fibers. Noting that a larger sample size and follow-up of more than one time point are needed, there was also a difference in the injury models used. The injury model in the ES study included transection of the sciatic nerve and repair by suture. In contrast, the MS study used an injury model of a 1 cm gap repaired by a reversed autograft (a relatively severe injury).

**Table 3:** Studies Included in the Analysis of Axonal Counts and Myelinated Fibre Counts on the Distal End of the Injury Following External Stimulation

Title	Mode of Stimulation	Follow-Up Period Studied	Sample Size	Control	Parameter	Control Result	Experiment Group Result	Relative Difference After Stimulation
Comparative effects of implanted electrodes with differing contact patterns on peripheral nerve regeneration and functional recovery^80^	Whole circle contact electrodes (Diameter = 2 mm) were placed 0.5 cm distal to the nerve suture site through the subcutaneous tunnel. Parameters: 20 Hz; Pulse width, 100 μs; 9 V; Waveform, square form. Electric stimulation of the nerve was conducted 30 min per day for 20 days in the four groups, starting on the first post-surgical day	4 and 10 weeks	6	No electrode, no ES	Myelinated distal fibre counts	4 weeks: 100 10 weeks: 110	4 weeks: 160 10 weeks: 240	4 weeks: 1.6 10 weeks: 2.1
Brief Electrical Stimulation Accelerates Axon Regeneration and Promotes Recovery Following Nerve Transection and Repair in Mice^77^	A stainless-steel electrode hooked around the sciatic nerve 2 mm proximal to the repair site—return electrode in subcutaneous tissue. Parameters: 16 Hz, 0.5 mA 10 mins or 60 mins	2 weeks	10	No ES, Uninjured	Myelinated axon count	Uninjured: 4,521 ± 468 No ES: 496 ± 218	835 ± 239	1.6
Bioluminescent optogenetics: A novel experimental therapy to promote axon regeneration after peripheral nerve injury^88^	Administration CTZ to activate BL-OG.	4 weeks	10 mice	Wild type(WT), untreated mice	Number of motoneurons (motor axon regeneration)	10	22 (in eLMO3 treatment)	2.2
Enhancing Motor and Sensory Axon Regeneration after Peripheral Nerve Injury Using Bioluminescent Optogenetics^87^	Bioluminescence, genetic delivery	4 weeks	8 mice	Contralateral nerve and negative untreated sciatic nerve	Number of motoneurons	Contralateral intact nerve; 52.1 negative control R115: 9	50.5 eLMO3 treatment	5.6 0.96
Optogenetically enhanced axon regeneration: motor versus sensory neuron-specific stimulation^89^	Pulses of blue light (473 nm) were shown directly onto the nerve via a fibre optic cable (200 μm)	4 weeks	7 mice	Untreated	Number of motoneurons	40.4 intact motoneurons	58.2 optically treated	1.44
Effect of the combination of high-frequency repetitive magnetic stimulation and neurotropic on injured sciatic nerve regeneration in rats^86^	Peripheral HFr-MS treatment (20 Hz, 20 min/d) was delivered ten consecutive days after autografting. HFr-MS was delivered using a 70 mm coil connected to a magnetic stimulator	17 days	8	The control group received only a reversed autograft in the left sciatic nerve with no treatment.	Number of myelinated fibers in the distal end	100	155	1.55
Stimulation and Repair of Peripheral Nerves Using Bioadhesive Graft-Antenna^90^	TMS delivered pulsed stimuli at a field magnitude of ≈0.72 T	8 weeks	5	Uncut nerve and chitosan adhesive without stimulation	Number of myelinated axons	1202 ± 66 (chitosan control)	1396 ± 68	1.16

Additionally, the number of axonal counts and motoneuron counts were analyzed. Five of the 19 studies that reported myelinated axons and motoneuron counts were identified for comparison (Table 3). The quantitative histological assessment showed that ES has the highest fold increase (1.6) in the myelinated axons on the distal end at an earlier time point compared with OS and MS. Optogenetic stimulation at 4 weeks showed a 1.5-fold increase, followed by 1.16 in MS. Although statistically significant, the increase in the fold change observed in MS was almost 4 times slower than ES, and its efficiency was 27.5% lower than ES (Fig. 2B). Compared with OS, MS was observed to be 4 times slower and 23% less effective. Electrical stimulation was found to be the fastest and most effective in this comparison.

**Figure 3:**
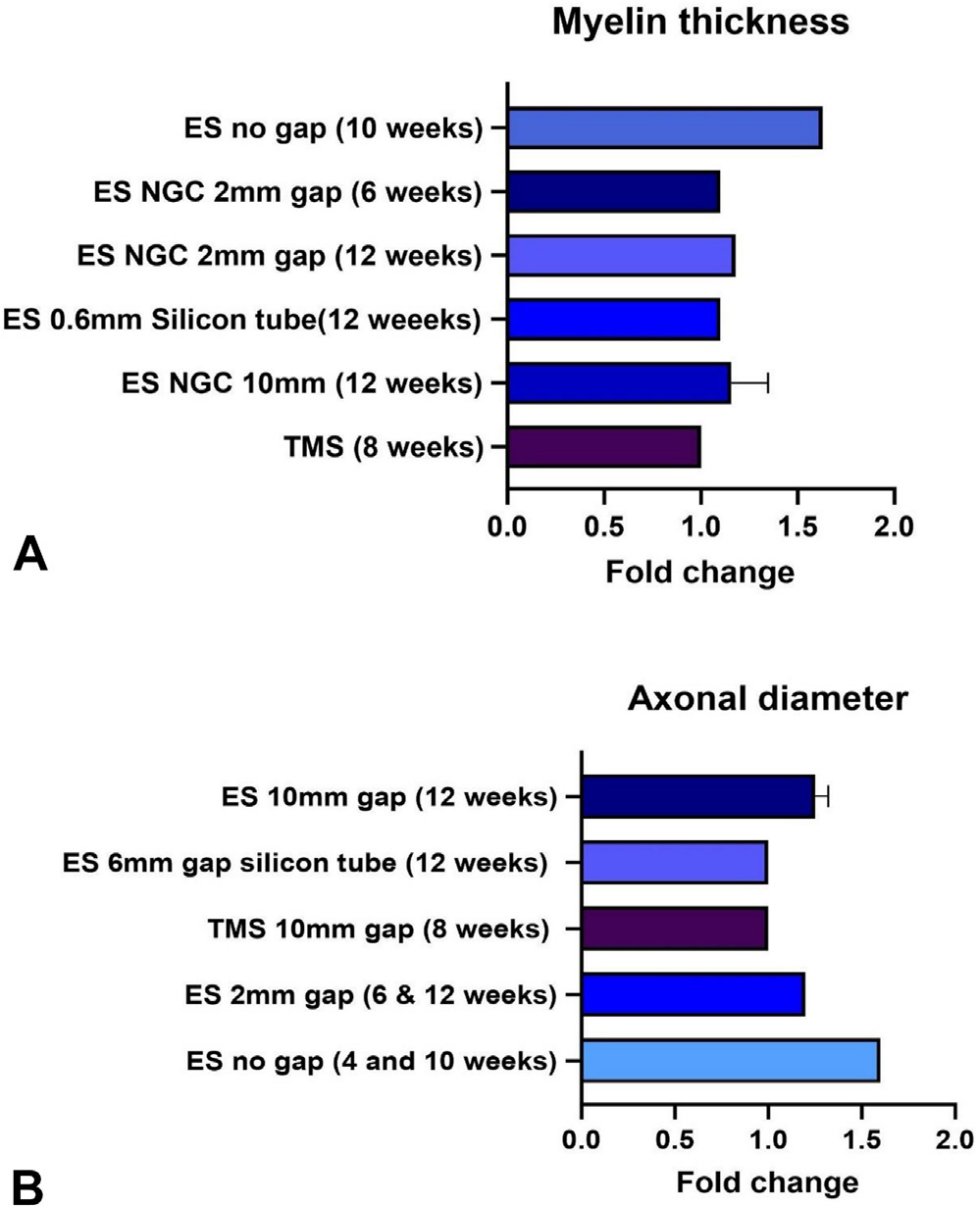
The improvement in histological features such as myelin thickness and axonal diameter following stimulations compared to no stimulation groups in rat/mouse models of sciatic nerve injury. **A** The improvement in the thickness of the myelin sheath after stimulations.^85^^,^^90^, ^92^, ^93^, ^94^, ^95^, ^96^**B** Fold change in the diameter of the regenerated axons after stimulations.^85^^,^^90^, ^92^, ^93^, ^94^, ^95^

Given that axonal and myelin fiber counts alone cannot indicate regeneration, it is imperative to include morphological aspects of the regenerating units, such as their diameters and the surrounding myelin thickness. Hence, axonal diameter (Table 4) and myelin thickness (Table 5) were analyzed to compare the effects of the stimulation. Seven studies were considered to compare the myelin thickness of the regenerative fibers in different stimulation groups, one of which used MS (TMS) (Fig. 3A). It was observed that TMS did not increase the myelin thickness significantly. In the case of ES, all the studies showed significant increases in myelin thickness, and ES performed better than TMS in improving myelin thickness. Next, the change in the diameter of regenerated axons was studied (Fig. 3B). The axonal diameter at 8 weeks in nerve repair models that underwent TMS was not significantly different from the control group, and its fold change was observed as 1 in the analysis. In the case of ES at 12 weeks, the fold increase was 1.2–1.3 in a 1 cm gap injury where conductive NGCs were used. The ES group with a 2 mm gap with follow-up times at 6 and 12 weeks also noticed an increase by 1.2-fold in the axonal diameter. As expected, a higher fold increase was seen in ES at 10 weeks in case of a no-gap injury or transection and suture repair. However, based on the severity of the injury, ES combined with conductive NGCs showed better axonal diameter recovery than all other groups.

**Table 4:** Studies Included in the Analysis of Fold Change in Axonal Diameter as a Histological Result of Stimulation

Title	Stimulation	Injury	Follow Up	Control	Sample Size	Axon Diameter of the Control Group (μm)	Axon Diameter of Experiment Group (μm)	Axon Diameter Improvement (Ex Diameter/Control Diameter)
Investigating the effects of brief electrical stimulation duration on sciatic nerve regeneration and functional recovery in a rat transection model^92^	Directly on nerve	6 mm gap repaired by a silicon tube.	12 weeks	No ES	6 rats per group of 10 min, 30 min, and 60 mins stimulation (Only considered 60 min group of data )	3.2	3.3	1 (No significant difference)
Conductive Composite Fibers with Optimized Alignment Guides Neural Regeneration under Electrical Stimulation^93^	Conduit, five times ES every other day	10 mm gap	12 weeks	No ES	3	2.8	3.45	1.2
Preparation of carboxylic graphene oxide-composite polypyrrole conduits and their effect on sciatic nerve repair under electrical Stimulation^91^	Conduit, ES 1 hour per day	10 mm gap	12 weeks	No ES	3	2.6	3.4	1.3
Comparative effects of implanted electrodes with differing contact patterns on peripheral nerve regeneration and functional recovery^85^	Cuff, direct stimulation	No gap, transection sutured	4 and 10 weeks	No ES	6	4 weeks: 1.5 10 weeks: 1.65	4 weeks: 2.45 10 weeks: 2.65	4 weeks: 1.6 10 weeks: 1.6
Bridging peripheral nerves using a diacetyl chitin conduit combined with short-term electrical stimulation^95^	Conduit, direct electrical stimulation	2 mm gap	6 and 12 weeks	No ES	8	6 weeks: 0.8 12 weeks: 0.82	6 weeks: 0.98 12 weeks: 1.0	6 weeks: 1.22 12 weeks: 1.21
Stimulation and Repair of Peripheral Nerves Using Bioadhesive Graft-Antenna^90^	TMS	10 mm isograft	8 weeks	No MS	3	3.1 ± 0.8	3.2 ± 0.8	No significant difference

**Table 5:** Studies Included in the Analysis of Fold Change in Myelin Thickness as a Result of Stimulation

Title	Stimulation	Injury	Follow Up	Control	Sample Size	Myelin Thickness of the Control Group (μm)	Myelin Thickness of Experiment Group (μm)	Myelin Thickness Improvement (Experiment Group/Control Group)
Investigating the effects of brief electrical stimulation duration on sciatic nerve regeneration and functional recovery in a rat transection model^92^	ES	6 mm sciatic nerve gap repaired by a silicon tube	12 weeks	No ES	6 rats per group of 10 min, 30 min, and 60 mins stimulation (Only considered 60 min group of data)	0.5	0.55	1.1
Conductive Composite Fibers with Optimized Alignment Guides Neural Regeneration under Electrical Stimulation^93^	ES	1 cm	12 weeks	No ES	3	0.48	0.65	No significant difference
Preparation of carboxylic graphene oxide-composite polypyrrole conduits and their effect on sciatic nerve repair under electrical stimulation^91^	ES	1 cm	12 weeks	No ES	3	0.39	0.43	1.1
Comparative effects of implanted electrodes with differing contact patterns on peripheral nerve regeneration and functional recovery^85^	ES	No gap, gap sutured	4 and 10 weeks	No ES	6	4 weeks: 0.25 10 weeks: 0.35	4 weeks: 0.4 10 weeks: 0.57	4 weeks: 1.6 10 weeks: 1.63
Bridging peripheral nerves using a diacetyl chitin conduit combined with short-term electrical stimulation^95^	ES	2 mm	6 and 12 weeks	No ES	8	6 weeks: 0.48 12 weeks: 0.5	6 weeks: 0.55 12 weeks: 0.59	6 weeks: 1.1 12 weeks: 1.18
Application of conductive Ppy/SF composite scaffold and electrical stimulation for neural tissue engineering^96^	Conduit, every two days, seven times	10 mm gap	12 weeks	No ES	3	0.45	0.62	1.37
Stimulation and Repair of Peripheral Nerves Using Bioadhesive Graft-Antenna^90^	TMS	10 mm isograft	8 weeks	No MS	5	2.0 ± 0.8	2.1 ± 0.8	No significant difference

### Electrophysiological outcomes

To compare the electrophysiological outcomes of different stimulations, CMAP or amplitude of CMAP, maximal distal muscle response (Mmax), and NCVs of the repaired nerves were studied in detail and compared.

**Figure 4:**
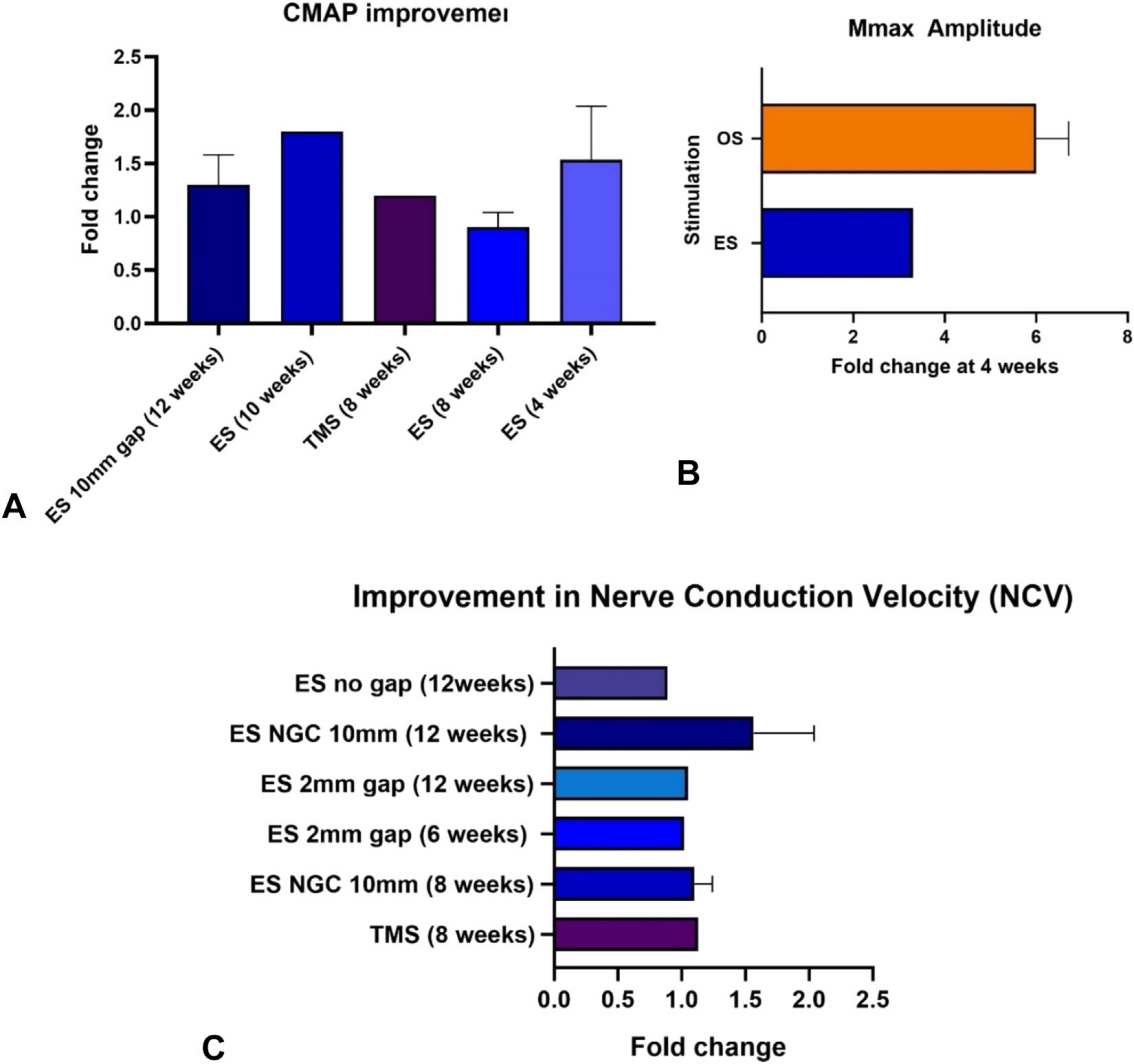
The comparison of electrophysiological improvement in nerve repair after using external stimulations compared to unstimulated controls in rat/mouse models. **A** Improvement of the compound muscle action potential (CMAP) or AMP on the distal end of the nerve injury after external stimulations.^85^^,^^90^^,^^94^^,^^98^^,^^99^**B** Mmax amplitude increases as fold changes after electrical and optogenetic stimulations.^87^^,^^88^^,^^100^**C** The improvement was measured as a fold change in nerve conduction velocity (NCV) after using different stimulations.^90^^,^^94^^,^^95^^,^^97^^,^^98^

Six studies reported the CMAP results from the data set. Compound muscle action potential and peak amplitude of CMAP have been used interchangeably (Table 6). In the case of TMS, it was observed that there was a 10% improvement in CMAP compared with groups devoid of stimulation. Electrical stimulation showed no significant difference in the same follow-up time of 8 weeks. At 56 days postsurgery, MS studies showcased 48% CMAP compared with contralateral nerves, indicating a significant enhancement in functional recovery (Fig. 4A).

**Table 6:** Studies Included From the Data Set to Compare the Fold Change to Indicate NCV When Using Different Stimulation for PNR

Title	Stimulation	Injury	Follow Up	Control	Sample Size	NCV of the Control Group	NCV of Experiment Group	NCV Improvement
Stimulation and repair of peripheral nerves using bioadhesive graft-antenna^90^	TMS	Sciatic nerve defect of 10 mm	8 weeks	Calculated as % of the contralateral nerve	5	36.656 ± 33	41.74 ± 4.13 ms^-1^	1.13
Preparation of carboxylic graphene oxide-composite polypyrrole conduits and their effect on sciatic nerve repair under electrical Stimulation^94^	ES	10 mm nerve gap	8,12 weeks (4 weeks data not included in analysis)	Autograft and no ES groups	3	8 weeks: 25 m/s 12 weeks: 28.06 m/s	8 weeks: 30 12 weeks: 34.59	8 weeks: 1.2 12 weeks: 1.23
Transcutaneous and direct electrical stimulation of mouse sciatic nerve accelerate functional recovery after nerve transection and immediate repair^97^	Only DES considered for analysis as results of TCES were not significantly different from DES	Transaction and repair	12 weeks	Axotomy	8	41.04 ± 6.14 ms	36.56 ± 5.88 TCES 36.61 ± 3.45	0.89
Bridging peripheral nerves using a deacetyl chitin conduit combined with short-term electrical stimulation^95^	ES	2 mm	6 weeks and 12 weeks	No ES	8	6 weeks: 41 m/s 12 weeks: 42 m/s	6 weeks: 42.5 12 weeks: 44.5	6 weeks: 1.02 12 weeks: 1.05
CNT/sericin conductive nerve guidance conduit promotes functional recovery of transected peripheral nerve injury in a rat model^98^	ES	10 mm nerve defect	12 weeks	Sham, autologous graft, No ES	6	8 weeks: 21 12 weeks: 20 m/s	8 weeks: 21 12 weeks: 38	8 weeks: 1 12 weeks: 1.9

The Mmax in the gastrocnemius muscle was compared (Table 7). The Mmax is the measure of synchronous activation of muscle fibers following a stimulation.^101^ Bioluminescence OS showed a Mmax improvement of 5.5-fold, where the transected nerve was repaired using fibrin glue (Fig. 4B). Another study that used bioluminescence OS to treat a 10 mm nerve gap showed a 6.5-fold increase in Mmax. Compared with OS, ES with transection (no-gap injury) showed improvement of 3.3-, 1.7-, and 1.7-fold at 4-, 8-, and 12-week follow-up, respectively. Optogenetic stimulation seems to outperform ES in terms of Mmax improvement (Fig. 4B).

**Table 7:** Studies Included for Comparison of CMAP or AMP as an Electrophysiological Parameter to Evaluate Outcomes of Stimulations

Title	Stimulation	Injury	Follow up	Control	Sample Size	CMAP of the Control Group	CMAP of Experiment Group	CMAP Improvement
Stimulation and repair of peripheral nerves using bio adhesive graft-antenna^85^	TMS	Sciatic nerve defect of 10 mm	8 weeks	Calculated as % of the contralateral nerve	5	38% (0.26 ± 0.09 mV)	48% (0.33 ± 0.09 mV)	10%
Multiple sessions of therapeutic electrical stimulation using implantable thin-film wireless nerve stimulators improve functional recovery after sciatic nerve isograft repair^99^	Six days ES	Isograft	2, 18 20 weeks	No stimulation (group 6 d considered due to its highest performance)	4	4 weeks: 4.25 mV 8 weeks: 5.75 mV 12 weeks: 8 mV 20 weeks: 5 mV	4 weeks: 3 mV 8 weeks: 4 mV 12 weeks: 8 mV 20 weeks: 11 mV	No significant difference
Comparative effects of implanted electrodes with different contact patterns on peripheral nerve regeneration and functional recovery^85^	ES full contact electrode	No gap	4, 10 weeks	No electrode	6	4 weeks: 3.5 mV 10 weeks: 5 mV	4 weeks: 7 mV 10 weeks: 9 mV	2 1.8
Conductive composite fibre with optimized alignment guides neural regeneration under electrical stimulation^99^	ES and conductive NGC	1 cm gap	12 weeks	Normal, Autograft, No ES	3	4 mV	6 mV	1.5
CNT/Sericin Conductive Nerve Guidance Conduit Promotes Functional Recovery of Transected Peripheral Nerve Injury in a Rat^98^	ES and conductive NGC	1 cm gap	8 weeks	Autograft, Sham, untreated	4	8 weeks: 2 mV 12 weeks: 1.8	8 weeks: 6 mV 12 weeks: 8 mV	8 weeks: 3 12 weeks: 4.4
Preparation of carboxylic graphene oxide -composite polypyrrole conduits and their effect on sciatic nerve repair under electrical stimulation^94^	ES and conductive NGC	10 mm nerve gap	4,8,12 weeks	Autologous, no ES	3	4 weeks: 1.4 mV 8 weeks: 2.7 mV 12 weeks: 2.5 mV	4 weeks: 2.3 mV 8 weeks: 2.4 mV 12 weeks: 2.96 mV	4 weeks: 1.6 8 weeks: 0.8 12 weeks: 1.1

Next, the NCV outcomes were compared across five studies (Table 8). It was observed that the NCVs of the repaired nerve significantly increased using both MS and ES (Fig. 4C). However, the fold change observed in the case of ES used along with an electroconductive NGC (with conductive carbon nanotubes) at 12 weeks was almost double that observed in other ES groups and TMS (Fig. 4C).

**Table 8:** Mmax- Maximal Direct Muscle Response in GAST. For the ES Studies Only 4-Week Data is Included in the Analysis

Title	Stimulation	Injury	Follow Up	Control	Sample Size	Mmax of the Control Group	Mmax of Experiment Group	Mmax Improvement
Bioluminescent Optogenetics: A Novel Experimental Therapy to Promote Axon Regeneration after Peripheral Nerve Injury^88^	Bioluminescence optogenetics	Transaction repair and fibrin glue	4 weeks	Scaled to WT expressed as fold	6	0.4	2.2	5.5
Enhancing Motor and Sensory Axon Regeneration after Peripheral Nerve Injury Using Bioluminescent Optogenetics^87^	Bioluminescence optogenetics	10 mm nerve gap	4,8,12 weeks	Mmax/intact	8	0.1	0.65	6.5
Effects of Repeated 20-Hz Electrical Stimulation on Functional Recovery Following Peripheral Nerve Injury^100^	ES	No gap, transaction	4 weeks	Mmax/intact	3	0.09	0.3	3.3

### Functional recovery

**Figure 5:**
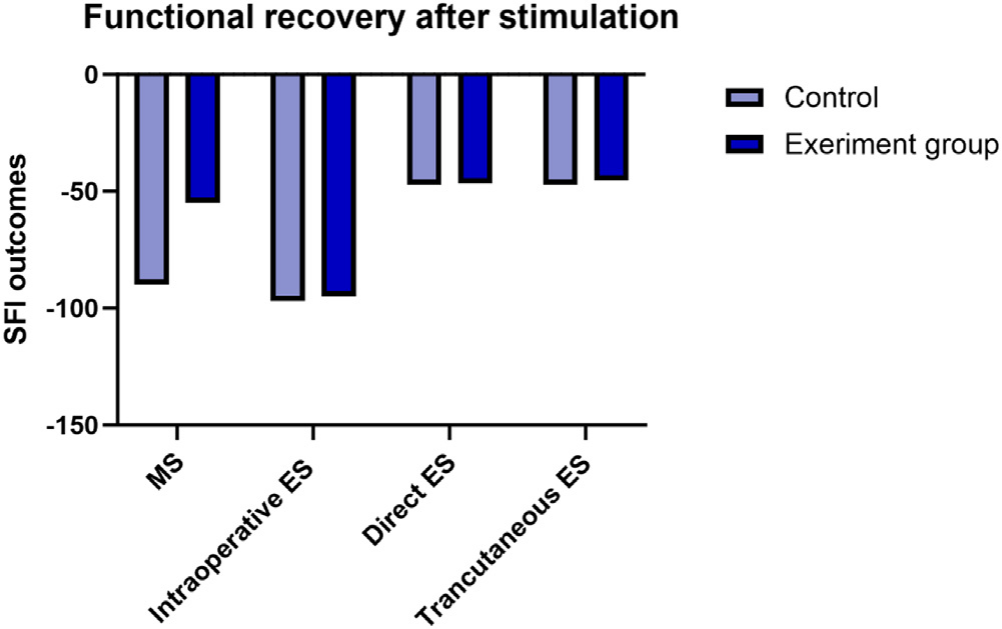
Effects of stimulation on the functional recovery in peripheral nerve repair measured using the Sciatic Function Index (SFI) at 2 weeks follow-up (ES) and 17-day follow-up (MS) in rat/mouse models.^86^^,^^97^^,^^99^

Three studies reported SFI outcomes across the 19 reviewed (Table 9). Although HFr-MS showed a 35% improvement in the SFI results at 17 days of follow-up, the ES group showed no significant improvement in SFI at the 2-week time point (Fig. 5).

**Table 9:** Studies Included in the Analysis of Functional Recovery Based on Relative SFI Improvement

Title	Stimulation	Injury	Follow Up	Control	Sample Size	SFI of the Control Group	SFI of Experiment Group	SFI Improvement
Effect of the combination of high-frequency repetitive magnetic stimulation and neurotrophin on injured sciatic nerve regeneration in rats^86^	Repetitive MS	Sciatic nerve transection and gap	17 days	Isograft	8	−90	−55	35% improvement
Effect of Intraoperative Electrical Stimulation on Recovery after Rat Sciatic Nerve Isograft Repair^99^	Intraoperative ES	Sciatic nerve transection	2 weeks	Isograft	40 rats	−97	−95	No significant difference between the groups
Transcutaneous and Direct Electrical Stimulation of Mouse Sciatic Nerve Accelerates Functional Recovery After Nerve Transection and Immediate Repair^97^	Direct ES (DES), Transcutaneous ES (TCES)	Sciatic nerve transection	2 weeks	Axotomy	28 mice	−47.07 ± 7;	DES: −46.58 ± TCES: - −45.45 ± 5.15	No significant difference between the groups

### Limitations

This review encountered challenges because of the scarcity of literature on MS and OS techniques. The data on functional analysis in OS and that of Mmax in MS were unavailable. The relative heterogeneous nature of some results underscores the necessity for standardized approaches and increased quantity and quality of research in MS and OS.

Additionally, the sample sizes of studies analyzed ranged between 3 and 8. In some instances, only two studies were compared (*n* = 1 in each group) to evaluate an improvement because of the lack of data and the shortage of studies. It was difficult to find studies with the same follow-up time points, and hence, it was impossible to compare many parameters at the same time points.

## Discussion

This comprehensive systematic review examines studies conducted between 2018 and 2023, focusing on ES, MS, and OS in PNR. Rigorous inclusion criteria led to the selection of 19 studies, with 13 concentrating on ES, 4 on OS, and 2 on MS strategies. The analysis categorized outcomes into quantitative histology, functional recovery, and electrophysiological evaluations, revealing significant challenges in comparing results because of diverse methodologies and variables influencing outcomes.

Histological assessments observed that HFr-MS and ES improved myelin fibers and axon counts. Interestingly, HFr-MS outperformed ES in myelin fiber counts on the distal ends and in the functional recovery analysis. Transcranial magnetic stimulation was observed to improve the regeneration significantly, but the improvement was slow and lower in magnitude than other stimulations. Optogenetic stimulation performed better than TMS in histological analysis and showed better outcomes than ES in electrophysiological analysis. Overall, ES demonstrated a consistent improvement in all the analyses. Interestingly, ES administered through conductive NGCs presented better results. High-frequency repetitive magnetic stimulation may be a viable alternative to ES in the future. However, larger data sets with uniform testing environments and parameters are essential. Despite not performing as well as the autograft in PNI treatments, external stimulation improved PNR, as inferred by the histomorphometry, electrophysiological, and functional analyses.

The study also identified gaps and limitations in our understanding of stimulation-enhanced nerve regeneration. There is a need for standardized stimulation protocols, incorporating longitudinal studies, and exploring combined stimulation approaches that could enhance reliability and comparability. Larger sample sizes and extended uniform follow-up periods will enable an accurate understanding of long-term effects. Although the findings provide crucial insights, translating them into clinical practice requires careful consideration. The limited number of clinical trials on external stimulation for PNR necessitates future studies addressing translational potential, focusing on safety, feasibility, and scalability in human applications based on in vivo models.

### Future perspective

Electrical stimulation has demonstrated its potential to expedite nerve regeneration. Innovations in ES delivery are crucial for enhancing its efficacy. An emerging trend involves the integration of conductive nanoparticles and polymers within NGCs to create better electrical conductivity and, consequently, more efficient neural interfaces.^102^^,^^103^ This improves stimulation delivery and develops tissue-engineered constructs.^104^, ^105^, ^106^ The main challenge with ES revolves around efficiently controlling the electrical conductivity of conductive polymers while varying their geometrical and morphological structures. Future research should focus on developing new synthetic methods and alignment techniques to enable large-scale production of conductive polymer nanomaterials.^106^, ^107^, ^108^ Electrode design and biocompatible material innovations would enhance selectivity and controllability, enabling more focal stimulation and reducing unintended side effects.^109^ Technological advances in electrode miniaturization and wireless control offer the potential for more convenient and patient-friendly neuroprosthetic devices. Addressing the clinical challenge of electrode stability over time will require research into long-term biocompatibility and integration with the host neural tissue.^110^

Magnetic stimulation offers a noninvasive alternative to ES. However, its clinical implementation remains constrained by the need for robust and expensive equipment.^8^^,^^111^^,^^112^ An emerging trend involves miniaturizing and optimizing MS devices to make them more accessible and patient-friendly.^113^^,^^114^ Contemporary trends have already begun revolving around designing compact, portable stimulators, making them feasible for in-home or point-of-care applications.^115^ Currently, most of the technology is still in preclinical investigation. Research into advanced coil designs and real-time magnetic resonance imaging–guided approaches aim to overcome the challenge of accurate targeting of MS.^116^, ^117^, ^118^, ^119^

Further research investigating the optimal strength and configuration of magnetic fields to ensure precise drug aggregation at nerve injury sites could stimulate further development of more sophisticated and portable magnetic field generators in vivo.^120^ Additionally, the ability to precisely target specific nerve regions and their functional interface remains a challenge and a focus for future research.^121^^,^^122^ This is especially an issue when the electrodes are placed in a flat surface interface, making MS difficult. Developing precise and controlled stimulation using coils within a similar diameter to peripheral nerves may be crucial in overcoming these limitations.

Optogenetic stimulation is an innovative technology that enables precise control of nerve cell activity with light. However, it faces significant challenges in clinical translation. The need for genetic modification presents an ethical consideration and a potential hurdle for clinical adoption. Future research should explore nongenetic strategies that achieve similar precision in nerve cell modulation. Furthermore, developing optogenetic tools compatible with human neural structures is essential, as many existing models use light wavelengths that do not penetrate deep enough into tissue.^123^^,^^124^ Modifying light sources and enhancing their wireless capabilities are emerging trends that may enable the clinical adoption of optogenetics.^125^ For instance, the integration of full-core optical fibers as waveguides, which intermediate the light transfer from external excitation sources toward the sample volume, has shown promise.^126^ This has led to the development of a system on a chip to model fluidic biointerfaces.^127^ Optogenetics in controlling neural activity further faces technical challenges in adapting the technology for clinical use. Nonviral vectors and chemical optogenetic modulators are emerging trends to alleviate these issues, eliminating the need for genetic alterations while offering spatiotemporal control of neural circuits.^128^, ^129^, ^130^ Moreover, optogenetic tools are refined to function in a wavelength range suitable for human tissues, allowing deeper penetration and more efficient stimulation of peripheral nerves.

In pursuing effective technologies for PNR, addressing technological and clinical challenges and fostering interdisciplinary collaborations is imperative. Emerging trends, such as incorporating conductive materials, miniaturizing devices, and nongenetic optogenetic strategies, hold promise in advancing these fields.

## Conflicts of Interest

No benefits in any form have been received or will be received related directly to this article.
